# e-Cigarette Tobacco Flavors, Public Health, and Toxicity: Narrative Review

**DOI:** 10.2196/51991

**Published:** 2024-05-27

**Authors:** Yehao Sun, Prital Prabhu, Ryan Rahman, Dongmei Li, Scott McIntosh, Irfan Rahman

**Affiliations:** 1 Department of Environmental Medicine University of Rochester Medical Center Rochester, NY United States; 2 Department of Clinical & Translational Research University of Rochester Medical Center Rochester, NY United States; 3 Department of Public Health Sciences University of Rochester Medical Center Rochester, NY United States

**Keywords:** vaping, e-cigarettes, tobacco flavors, toxicity, regulation, tobacco, public health, smoking, menthol, social media, nicotine, symptoms, symptom, risk, risks, toxicology, health risk, regulation

## Abstract

**Background:**

Recently, the US Food and Drug Administration implemented enforcement priorities against all flavored, cartridge-based e-cigarettes other than menthol and tobacco flavors. This ban undermined the products’ appeal to vapers, so e-cigarette manufacturers added flavorants of other attractive flavors into tobacco-flavored e-cigarettes and reestablished appeal.

**Objective:**

This review aims to analyze the impact of the addition of other flavorants in tobacco-flavored e-cigarettes on both human and public health issues and to propose further research as well as potential interventions.

**Methods:**

Searches for relevant literature published between 2018 and 2023 were performed. Cited articles about the toxicity of e-cigarette chemicals included those published before 2018, and governmental websites and documents were also included for crucial information.

**Results:**

Both the sales of e-cigarettes and posts on social media suggested that the manufacturers’ strategy was successful. The reestablished appeal causes not only a public health issue but also threats to the health of individual vapers. Research has shown an increase in toxicity associated with the flavorants commonly used in flavored e-cigarettes, which are likely added to tobacco-flavored e-cigarettes based on tobacco-derived and synthetic tobacco-free nicotine, and these other flavors are associated with higher clinical symptoms not often induced solely by natural, traditional tobacco flavors.

**Conclusions:**

The additional health risks posed by the flavorants are pronounced even without considering the toxicological interactions of the different tobacco flavorants, and more research should be done to understand the health risks thoroughly and to take proper actions accordingly for the regulation of these emerging products.

## Introduction

### Background

Tobacco flavoring is added to e-cigarettes to make them appealing to vapers, specifically by mimicking the taste of traditional cigarettes. Tobacco-flavored e-cigarettes are often advertised as a safer alternative to traditional cigarettes, which allow smokers to enjoy the taste they are familiar with more conveniently and smoothly for harm reduction. Tobacco-flavored e-cigarettes are very popular among various subpopulations of adults in the United States, with around 30% of vapers using these products [1]. However, the prevalence seems to be lower in dual users (vapers who also use traditional cigarettes) and vapers who used e-cigarettes as an attempt to quit smoking, with the percentages being 28.5% and 20.5%, respectively [2,3].

Although the taste of tobacco-flavored e-cigarettes mimics that of traditional cigarettes, the type of nicotine they contain may differ from traditional cigarettes. Recently, e-cigarette products have begun to contain synthetic nicotine or tobacco-free nicotine (TFN), a racemic mixture of both R- and S-nicotine isomers, which is different from the traditionally used tobacco-derived nicotine that is composed of pure S-nicotine [4]. Initially, e-cigarette products began to use TFN since it was not regulated by the US Food and Drug Administration (FDA), and products were able to be brought to the market since these products did not need to go through the premarket tobacco product application for e-cigarettes [4]. Although initially brought to the market without government regulation, in 2022, new legislation expanded the authority of the FDA to regulate TFN products as well [5]. Currently, limited data are available regarding the health effects of TFN, but studies have found that messaging by e-cigarette companies leads to the belief by e-cigarette users that TFN has a lower health risk compared to tobacco-derived nicotine and a higher intention to use TFN products [6]. Young adults between 18 and 25 years of age who were interested in trying TFN believed it to be less addictive than those who were uninterested, and those who have tried TFN reported that TFN products have flavors that taste better and smoother [7]. Similarly, young adults (aged 18-25 years) who were likely to purchase TFN pouches believed that TFN pouches were less harmful to a person’s health; less addictive; and tastes smoother, cleaner, and better compared to young adults who would not purchase TFN pouches [8]. Due to the perception in young adults that TFN is less harmful and addictive, there is a need for more research on the health effects of exposure to TFN to aid government regulation and properly educate the public about any potential risks of using these compounds. More recently, Nixodine (ie, (S)-6-methyl nicotine), which is a structural analog of nicotine, and synthetic-free nicotine or tobacco-free nicotine have been introduced into the market as well without much being known about their biological or toxicological effects.

Besides the use of TFN in tobacco-flavored e-cigarettes, another important modification to these products is the addition of flavorants commonly used in other flavors. On February 6, 2020, the FDA implemented enforcement priorities against all flavored, cartridge-based e-cigarettes other than tobacco- and menthol-flavored products [9]. According to Rostron et al [10] in 2020, as much as 93.2% of youth vapers started vaping with a flavored e-cigarette, and among those who are still vaping, 71% reported the flavors of e-cigarettes as a reason for use. It was also indicated that youth vapers preferred fruit and mint flavors to tobacco or menthol flavors [11]. The tobacco flavors of e-cigarettes are made to mimic the flavor of traditional tobacco cigarettes with some variation. There are many different tobacco flavors made from hundreds of brands that can provide the user with different types of tobacco flavors, including “Classic Tobacco,” “Smooth/Bold Tobacco,” and “Virginia Tobacco.” Demographically, tobacco flavors are more popular among adults and less popular among youth [12]. The lack of appeal of tobacco-flavored e-cigarettes to youth allows for fewer regulations. Therefore, we perceived that the ban on flavors other than tobacco and menthol undermined the e-cigarette products’ appeal to youth vapers, as their favorite flavors were removed from access, thus largely decreasing the manufacturers’ profit. To reverse the impacts brought by the difference in regulations, e-cigarette manufacturers started to blend other flavors into tobacco-flavored e-cigarettes, recreating the appeal for youth vapers [13,14]. For example, we found that an e-cigarette manufacturer has a fourth-generation e-cigarette product with a “Smooth Tobacco” flavor, which contains a combination of tobacco and cream flavors. The same entity also sells an e-liquid of “Tobacco Salt Rich” flavor, which is a mixture of tobacco, smokey vanilla, and creamy caramel flavors. Studies have also extracted flavorants that represent sweets and caramel-like flavors in an e-liquid marked “Smooth & Mild Tobacco” and multiple flavorants that do not belong to tobacco flavors in another tobacco-flavored e-liquid that was deidentified [13,15,16]. Such compounds include ethyl maltol, vanillin, corylone, and ethyl vanillin, which can lead to adverse health effects [14]. Additionally, the volatile organic compounds (VOCs), reactive oxygen species, and other compounds present in the tobacco-flavored e-liquids can pose further health risks.

### Objective

**Figure 1:**
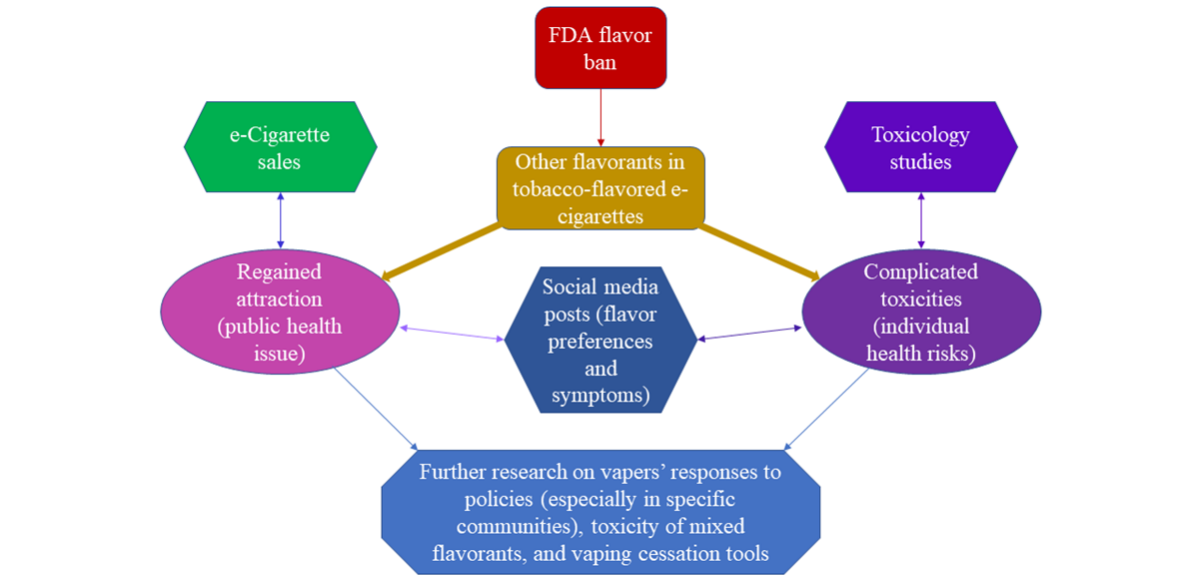
A schematic of the discussion of new tobacco-flavored e-cigarettes and their associated problems. FDA: Food and Drug Administration.

The emergence of these new tobacco flavors may serve as a source for public health issues, and information related to them is critical for the establishment of regulations and interventions. Therefore, by analyzing the toxicity, characteristics, sales, social media perception, and public health aspects of tobacco-flavored e-cigarettes, this review aims to inform authorities about this issue and provide information for potential interventions (Figure 1).

## Methods

To collect data, searches were conducted on Google Scholar and PubMed for papers published between 2018 and 2023 related to e-cigarette use patterns, toxicity of e-cigarette chemicals, social media posts about e-cigarettes, and public health interventions regarding e-cigarettes. Toxicity information was also included from articles published before 2018, and e-cigarette sales data and related policies were extracted from government websites and documents. The keywords for searching these sources of information included “tobacco-flavored e-cigarettes,” “e-cigarette use,” “synthetic nicotine,” “flavorants,” “e-cigarette policy,” “social media and vaping,” “vaping cessation,” and chemical names mentioned in this review.

The extracted information was discussed to identify the appeal of tobacco-flavored e-cigarettes based on sales data, to document the toxicity complications from toxicology studies, and to confirm the impacts of the addition of other flavorants in tobacco-flavored e-cigarettes by analyzing studies on related social media posts (Figure 1).

## Results

### e-Liquid Constituents Inhaled During Vaping

Tobacco-flavored e-cigarettes have a wide range of chemicals in the e-liquid, and different tobacco flavors have different flavoring agents. However, in general, tobacco-flavored e-cigarettes contain propylene glycol, glycerol, and 0-50 mg/mL of nicotine (in the form of freebase nicotine or nicotine salts), similar to most other e-cigarettes. Tobacco-flavored e-cigarettes have also been shown to have cinnamaldehyde [17]. Additionally, for the popular brands JUUL and Puff Bar, many other chemicals were frequently found to be in their tobacco-flavored e-cigarettes in greater than 1 mg/mL concentrations, including ethyl maltol, corylone, vanillin, and ethyl vanillin [14]. Another study found caffeine, isophorone, tributyl O-acetylcitrate, tributylphosphine oxide, triethyl citrate, and vanillin in tobacco-flavored e-liquids from popular brands such as JUUL, Blu, Smok, and Vuse Alto [18]. There are also many VOCs present in tobacco flavors such as ethanol, toluene, ethylbenzene, and styrene [17]. Moreover, tobacco flavors would also produce reactive oxygen species that cause oxidative stress when used. Overall, there are many different carbonyls, citrates, phenols, VOCs, and other organic compounds present in tobacco-flavored e-liquids and their combustion and degradation products that are inhaled during vaping.

### Cellular Toxicities of Tobacco-Flavored e-Cigarette Aerosols

Existing studies have established some knowledge of the toxicities of tobacco-flavored e-cigarettes [16,19-24]. The compounds present in the e-liquid and aerosol of tobacco-flavored e-cigarettes have many toxic effects on cells. For instance, nicotine in tobacco flavors can induce mucus hypersecretion by goblet cells and decrease mucociliary clearance in the lung by suppressing α7 nicotinic acetylcholine receptor activity and cystic fibrosis transmembrane conductance regulators, resulting in a greater risk for chronic lung diseases [19]. It was revealed that tobacco flavorants can induce oxidative stress, inflammation, DNA damage, and higher levels of cell death in lung epithelial cells and inflammatory responses in different types of cells including fibroblasts [20,21]. Overall, reported in either in vivo or in vitro studies, increased reactive oxygen species or oxidative stress and the release of inflammatory cytokines were associated with tobacco flavors, and the conclusions included increased cell death, decreased cell viability, and increased inflammatory responses [22].

### Mechanisms of Disease Pathogenesis Related to Toxicities of Tobacco-Flavored e-Cigarette Aerosol

Beyond cells, tobacco-flavored e-cigarettes are harmful to the user's overall health. Inhaling nicotine from tobacco-flavored e-cigarettes can result in hypertension, chronic obstructive pulmonary disease (COPD), increased myocardial infraction risk, and asthma [23]. The propylene glycol found in tobacco-flavored e-cigarettes can also pose health risks when inhaled, where cough, difficulty breathing, and increased asthma risk are linked to the inhalation of propylene glycol [23]. Moreover, the heating of glycerol found in tobacco-flavored e-liquids can produce formaldehyde, which can act as a carcinogen when inhaled [23]. In another study, it is also shown that tobacco flavor accompanied by the presence of nicotine can induce an allergic inflammatory response, characterized by elevated levels of eotaxin, interleukin-6, and chemokine (C-C motif) ligand 5 (also known as RANTES) [16]. The combination can also increase the level of plasminogen activator inhibitor-1, with a higher level being a risk factor for thrombosis and atherosclerosis [16,24]. Additionally, the reactive oxygen species and VOCs present in tobacco-flavored e-cigarettes can increase exposure to free radicals, resulting in oxidative stress and lung inflammation [19]. Overall, the inhalation of compounds present in tobacco-flavored e-cigarettes poses a serious health risk and can increase lung toxicity and the likelihood of various chronic lung diseases ranging from COPD to cardiovascular disease (Figure 1).

### Tobacco-Flavored e-Cigarette Products

Although the flavors are limited to tobacco flavors, there is still a variety of e-cigarette devices with distinct characteristics associated with tobacco flavors [25,26]. Generally, e-cigarette devices are divided into 4 generations, all of which can support tobacco flavors [26].

First-generation e-cigarettes are designed to mimic the appearance of traditional cigarettes and thus are also known as cig-a-likes [25,26]. The major components are a battery, an atomizing unit, and a fluid reservoir (cartridge) [26]. Although outdated, tobacco-flavored e-cigarettes of the first generation can still be found in some web-based and physical vape shops.

In second-generation e-cigarettes, the cartridge is replaced by a “clearomizer” installed in a pen-shaped device, so second-generation e-cigarettes are also called “vape pens” [25,26]. Third-generation e-cigarettes, on the other hand, are highly customizable and contain sub-ohm tanks, which allow even higher wattage due to decreased resistance [25,26]. Both second- and third-generation e-cigarettes use e-liquids for aerosol generation, and tobacco-flavored e-liquids can be easily found in web-based vape shops and are sold in large amounts.

Fourth-generation e-cigarettes are called “Pod-Mods,” indicating a modifiable pod cartridge that can be in various shapes [25,26]. Fourth-generation e-cigarettes use nicotine salts instead of the freebase nicotine used in previous generations, allowing a higher concentration of nicotine to be present [25]. A popular variation named “vape bars” is the most popular product in web-based vape shops.

Tobacco-flavored products associated with all the generations discussed above are widely available web-based vape shops for vapers, and the products are sold in large amounts. In web-based vape shops, the best-selling tobacco-flavored e-cigarette products are mostly vape bars (fourth-generation devices), followed by tobacco-flavored e-liquids (used by second- and third-generation devices). First-generation products and prefilled cartridges or pods (second-generation products) can also be found in another vape shop, where it claims that the first-generation product is “the new #1 selling e-cigarette on the market.” The vape shop selling primarily fourth-generation e-cigarettes has a better website design with different fonts that may attract young vapers, whereas the vape shop website that sells first- and second-generation e-cigarettes looks relatively old.

### Public Perceptions of Tobacco-Flavored e-Cigarettes on Social Media

An examination of the public perceptions of different e-liquid flavors on over 2 million e-cigarette–related Twitter (subsequently rebranded as X) posts from May 31 to August 22, 2019, showed the public had a more negative attitude toward the tobacco flavor (sentiment score=–0.134) using sentiment analysis [27]. Meanwhile, it was also found that the public was positive toward fruit (sentiment score=0.074) and sweets flavors (sentiment score=0.156), and most of the discussions were about these 2 flavors (58.15% and 14.67%, respectively) [27]. Immediately after the flavor ban, only menthol and tobacco flavors were allowed on the market, and an increase in discussion about menthol flavors (from 16.4% to 37%) was observed [9,28]. However, there was no significant increase in discussion about tobacco flavors, indicating that vapers likely did not choose to shift to tobacco flavors immediately after the ban of their favorite flavors [28]. In contrast, the discussion of fruit and sweets flavors remained high after the ban and even increased around 5 months later (from 41% and 22.3% before the ban to 57% and 28% five months after the ban, respectively), signaling that the vapers might have sought other sources for their favorite flavors after they were banned, which indicates continued interest in these flavors [28].

Through applying generalized estimating equation (GEE) models on over 3000 Reddit posts from January 2013 to April 2019 that comention e-cigarette use and health symptoms in the same Reddit post, it was found that tobacco flavor was more likely to be comentioned with respiratory and throat symptoms than other symptoms [29]. A specific examination of the JUUL pod tobacco flavor with health symptoms, using similar GEE models and Reddit posts from September 2016 to April 2019, showed a high probability of the comention of the JUUL tobacco flavor with throat, respiratory, and cardiovascular symptoms [30].

### e-Cigarette Sales After Flavor Ban Regulations and Flavorants’ Appeal to Vapers

The vast variety of e-cigarette flavorings, such as banana, mango, and cotton candy, are extremely appealing to the younger generation, which supports the nicotine addiction epidemic among today’s youth. However, the February 2020 FDA ban on flavored prefilled e-cigarette cartridges, while having the intention of curbing flavored e-cigarette use, also opened new doors for the vaping industry to continue making profits [9]. This was due to 2 loopholes in the FDA policy: the ban did not cover the sale of tobacco- and menthol-flavored prefilled cartridges or the sale of flavored disposable e-cigarettes [31]. For these reasons, e-cigarette users were able to find alternatives to flavored prefilled cartridges, such as the tobacco-flavored e-cigarettes outlined in this paper.

Centers for Disease Control and Prevention data show that after the FDA policy enactment, the unit share of disposable e-cigarettes went from 29.9% to 49.6%, whereas the respective unit share of prefilled cartridges lowered from 70% to 50.3% between February 2020 and July 2022 [31]. This data show the popularity of flavored e-cigarettes in the vaping population, with them quickly switching to disposable e-cigarettes once flavored prefilled cartridges became unavailable. Additionally, although the FDA ban was supposed to limit prefilled cartridge manufacturers such as JUUL from profiting off of nicotine addiction, it allowed disposable vaping brands, such as Puff Bar, Elf Bar, and Blu, to achieve a massive increase in sales by developing products that filled the “flavoring hole” left by the prefilled cartridge ban. Data showed that in response to these holes, e-cigarette users largely switched to disposable devices rather than continuing to buy the tobacco- and menthol-flavored cartridges still on the market [31]. After the 2020 ban up until July 2022, tobacco-flavored cartridge sales only increased by 11.9%, whereas all other flavor sales increased by 75.6% [31], showing the preference of the vaping population for nontobacco flavorings, which indicates that vapers are likely to be attracted to the new tobacco flavors that contain flavorants from other flavors.

### Public Health Interventions Associated With Tobacco Flavors

Flavors have been cited as a key factor for the initiation of vaping by adolescents and young persons and facilitate the ongoing use of vaping products by those of all ages. Flavored vaping products are alluring to both new and established tobacco product users, and a wide variety of flavors are available. This wide variety and the ability to combine different flavors, in this case, the addition of other flavorants into tobacco flavors, could contribute to the ongoing vaping behavior among both youth and adults [12,32].

Per the FDA “Deeming” regulations, the FDA can now regulate the presence and amount of “characterizing flavors” in vaping products [33]. According to former FDA Commissioner Dr. Scott Gottlieb, e-cigarette use among youth can be characterized as an epidemic [34]. Users must be at least 18 years of age to buy vaping products in most states, but those younger than 18 years old are still able to purchase from a variety of retailers and web-based vendors [12,33].

To address the vaping epidemic, especially among youth, in 2021, the FDA implemented a flavor enforcement policy to restrict the sales of all cartridge-based, unauthorized, flavored e-cigarettes other than tobacco and menthol flavors [35,36]. Evaluation of the impact of the FDA flavor enforcement policy on e-cigarette use behavior is in progress. One study assessed the potential impact of the flavor enforcement policy on a specific vaping-related behavior change—quitting vaping—using natural language processing strategies with data collected from the Twitter platform [35]. The proportion of tweets (and Twitter users’ mentions) concerning quitting vaping was compared before and after the implementation of the FDA flavor policy [35]. Compared to before the FDA flavor policy, the proportion of tweets and Twitter user mentions after the implementation of the policy was higher [35]. They also reported that after the policy implementation (compared to before), there was an increasing trend in the proportion of female individuals and young adults (18-35 years old) mentioning quitting vaping [35]. They concluded that, as observed on Twitter, the FDA policy did have a positive effect on vaping cessation and therefore a potential influence on broader definitions of vaping behavior [35].

Another public health intervention for vaping cessation is the use of free vaping cessation apps, which have various content, features, and adherence to evidence-based approaches. In 2020, researchers conducted a systematic search of existing smartphone apps for vaping cessation [37]. A total of 8 apps were included in a quality assessment and content analysis. They concluded that the few existing vaping cessation apps use similar approaches to smoking cessation apps but are potentially valuable tools [37].

## Discussion

### Toxicological Complexities Brought by the Addition of Other Flavorants

Besides the toxicities of tobacco-flavored e-cigarette constituents, the introduction of other previously irrelevant chemicals may inevitably complicate the toxicity of these products. The most commonly used flavorant (in 35% of e-liquids), vanillin, is responsible for vanilla flavors in e-liquids; is likely to be present in the “Tobacco Salt Rich” e-cigarette; and was extracted from the deidentified tobacco-flavored e-cigarette introduced earlier in this paper [16,38,39]. As shown, the presence of vanillin has a positive correlation with the toxicity of e-liquids (*R*^2^=0.62) [38]. The vanillin in tobacco flavors is inflammatory and can irritate airways [19]. Another popular flavorant (in 32% of e-liquids) present in caramel flavors, ethyl maltol, was also present in the deidentified tobacco-flavored e-cigarette and has been shown to be a contributing factor for incidences of kidney lesions in rats and mild hemolytic anemia in dogs [15,16,39,40]. Furthermore, the inhalation of cinnamaldehyde and ethyl maltol, compounds found in tobacco flavors, causes oxidative stress and can lead to inflammation and epithelial barrier dysfunction, increasing the risk of diseases such as COPD [19]. These are only 2 of the flavorants used in e-liquids, and the typical number of different flavorants in a single e-liquid product would be higher than 10 [38]. It was found that the more chemicals there are in the e-liquid, the higher the toxicity that the e-liquid is likely to possess [38]. Therefore, it is predicted that the additional flavorants in tobacco-flavored e-cigarettes, which already contained many kinds of flavorants, would increase the overall toxicity of the product, and it would be hard to figure out the interactions of the toxicity mechanisms related to flavorants that originally belonged to completely unrelated species. More studies are required to fully understand this complexity and take appropriate actions regarding the regulation.

### Youth Vapers’ Preferences

According to scientific studies, e-cigarette users’ preferences for e-cigarette devices were shifting toward newer-generation devices: Fourth-generation devices (prefilled pod cartridges) are the most used devices, although third-generation devices still take up a considerable proportion of use [41]. It was also observed that the shift toward newer generations is faster in youth users than in young adults or older adults [41]. Another study conducted by Lin et al [42] also agrees with this finding, as they found that adolescent and young adults’ preference is responsive to advancements in e-cigarette technology. They generally avoid using earlier-generation devices (the percentage of users who usually used disposable or large-size rechargeable e-cigarettes dropped from 88.2% to 33.1% during the study) and prefer more innovative products (the percentage of users who usually used pod-based e-cigarettes, which were only introduced into the market when the study was halfway through, was 22.3% by the end of the study) [42]. The trend found by those studies is likely applicable to tobacco-flavored e-cigarettes, as the characteristics of web-based vape shops discussed above match the trend [41,42]. The fact that youth vapers shifted to pod-based e-cigarettes quickly also made the addition of other flavorants in these products a more significant public health issue.

### Implications From Social Media Studies

According to results from the social media studies mentioned earlier, vapers demonstrated continued interest in fruit and sweets flavors immediately after the flavor ban while remaining uninterested in original tobacco flavors [27,28]. Our web-based survey study also showed that most vapers continued using flavored e-cigarettes even after the flavor ban, as disposable e-cigarettes were not covered by the FDA flavor ban [43]. Therefore, when their favorite flavors are integrated back into tobacco flavors, it is expected that they would prefer the mixed flavor. Since the availability of flavors was among the top reasons for vaping and its initiation, especially in adolescents and young adults, the addition of these flavors in tobacco flavors would likely resuscitate the motivation for vapers to continue to vape [44,45].

Social media research that focused on health issues comentioned with flavors discovered that tobacco flavors were generally more likely to be comentioned with respiratory and throat symptoms, and cardiovascular symptoms were also frequently comentioned if the tobacco-flavored e-cigarettes were from JUUL [29,30]. These results are associated with traditional, tobacco-flavored cigarettes prior to the addition of new flavors, and the addition might be associated with more complicated symptoms. In the web-based vape shop, we found that for new tobacco-flavored e-cigarettes, the best-selling ones often contained new flavors categorized as “sweets” flavors or the “crème” flavor in JUUL products [29,30]. According to the same GEE models, “sweets” flavors were comentioned with throat and digestive symptoms, whereas JUUL’s “crème” flavor was comentioned with neurological, digestive, and “other” symptoms, which were not observed in the corresponding tobacco flavors [29,30]. However, the comention of flavors with health symptoms does not indicate that vaping will cause these symptoms, as it is also possible that vaping could reduce the health symptoms. Previous study showed that the toxicological effects of the flavorants may interact with each other, and the effects of such interactions are unknown [46]. Therefore, more research should be done to further understand the symptoms associated with the addition of other flavors into tobacco-flavored e-cigarettes.

Overall, as we observed more varieties of tobacco-flavored e-cigarettes sold in vape shops, the public perceptions of tobacco-flavored e-cigarettes and their associations with health symptoms mentioned on social media need to be revisited.

### Vaping Communities and Flavor Addition to Tobacco Flavors

Since vapers can belong to a variety of different communities, the addition of other flavors into tobacco-flavored e-cigarettes may have different effects in these communities, and we need to focus on the differences. For example, the vaping behaviors of dual users of both traditional cigarettes and e-cigarettes are different from vapers who only use e-cigarettes [2]. Dual users usually only use e-cigarettes when they are engaging in activities or in places that encourage e-cigarette use, or when they use e-cigarettes as substitutes for traditional cigarettes [47]. This type of difference becomes exceedingly important when there is a relatively high prevalence of vaping in the community (including minority youth) or when the community is our major target of protection (including age groups such as adolescents) [48]. For instance, among young adult e-cigarette users, bisexual women were the most susceptible to e-cigarette use habits with a high level of cigarette use [49]. Such disparities are of importance when public health interventions are tailored, so knowing how specific communities respond to the addition of flavorants in tobacco-flavored e-cigarettes is critical. However, despite this importance, there is minimal data on this issue, and the differential effects remain unknown to us. Further studies should be done on these specific communities for us to comprehensively understand how new tobacco-flavored e-cigarettes impact the entire vaping population and establish regulations accordingly.

### Conclusion

After the FDA implemented enforcement priorities against all flavored, cartridge-based e-cigarettes other than tobacco- and menthol-flavored products on February 6, 2020, most e-cigarette products became regulated, leaving only menthol and tobacco flavors to be widely and legally available for vapers [9]. This ban on other flavors impaired e-cigarettes’ appeal to vapers, so e-cigarette manufacturers decided to recreate similar flavors by blending the corresponding flavorants into tobacco-flavored e-cigarettes to form variant tobacco flavors, including “Smooth Tobacco” [13,16]. These mixed tobacco flavors are now widely available in web-based vape shops, and the products either come as or can be used in any generation of e-cigarettes to accommodate the preference of vapers in different age groups (it is inferred that younger vapers’ preferences switch to more innovative products more easily and they generally use newer-generation devices) [41,42].

Evidence from both the vaping market share and social media posts indicate that the manufacturers’ strategy was successful [28,31]. After the FDA regulation, the unit share of prefilled cartridges decreased, and the sales of disposable e-cigarettes of flavors other than tobacco flavors increased dramatically, indicating a strong preference for flavorants in other flavors that motivated the vapers to switch to disposable e-cigarettes [31]. Therefore, the addition of these flavorants into tobacco flavors may establish appeal to the new tobacco flavors. On the other hand, similar trends were found in social media posts, showing that fruit and sweets flavors were still often discussed after the flavor ban policies [28]. The heated discussions indicate the vapers’ strong craving for these flavors, so this further confirms that the addition of other flavorants into tobacco flavors may successfully attract vapers.

This strategy by the manufacturers can not only lead to new public health issues but also new health risks and symptoms in individual users, and it even raises issues on harm reduction approaches. The additional flavorants mixed in the new tobacco-flavored e-cigarettes may have unique toxicology mechanisms that are not observed in flavorants used in traditional tobacco flavors. For example, vanillin and ethyl maltol are likely found in a product with the flavor “Tobacco Salt Rich” and another deidentified tobacco-flavored e-cigarette, and these flavorants have been shown to increase the toxicity of e-liquids and induce incidences of kidney lesions in rats and mild hemolytic anemia in dogs, respectively [15,16,38-40]. Other flavorants may also be integrated into the recipe of tobacco-flavored e-cigarettes, and it has been shown that the toxicity of the e-liquids increases as the number of chemicals in its recipe increases [38]. Meanwhile, in the analysis of Reddit posts using GEE models, the “sweets” flavors in e-cigarettes were associated with higher comention of digestive and throat symptoms, which are not demonstrated in traditional tobacco flavors [29]. Therefore, the symptoms associated with e-cigarette use are likely to be more complicated when using the new tobacco-flavored e-cigarettes. However, our predictions of toxicology and symptoms are based on the simple addition of effects, where the interactions between the flavorants were not taken into consideration. More research needs to be done to fully understand the interactions and the overall effects.

Besides the public health issues and personal health risks associated with the addition of flavorants in tobacco-flavored e-cigarettes, the FDA flavor ban policies did have an overall positive effect in helping vapers quit vaping [36]. The use of the new vaping cessation apps is also a potentially important aspect of public health interventions [37]. To further extend the positive effects, more research should be done to analyze the effects brought by the manufacturers’ efforts to bypass the regulations and premarketing approval, and emphasis should be placed on vulnerable communities regarding the vaping public health effects.

## Abbreviations

COPD: chronic obstructive pulmonary disease
FDA: Food and Drug Administration
GEE: generalized estimating equation
TFN: tobacco-free nicotine
VOC: volatile organic compound

## References

[1] Leventhal AM, Dai H (2021). Prevalence of flavored e-cigarette use among subpopulations of adults in the United States. J Natl Cancer Inst.

[2] Zavala-Arciniega L, Hirschtick JL, Meza R, Fleischer NL (2022). e-Cigarette characteristics and cigarette smoking cessation behaviors among U.S. adult dual users of cigarettes and e-cigarettes. Prev Med Rep.

[3] Bold K, O'Malley S, Krishnan-Sarin S, Morean M (2023). e-Cigarette use patterns, flavors, and device characteristics associated with quitting smoking among a U.S. sample of adults using e-cigarettes in a smoking cessation attempt. Nicotine Tob Res.

[4] Jordt SE (2023). Synthetic nicotine has arrived. Tob Control.

[5] Stephenson J (2022). FDA gains power to regulate synthetic nicotine in e-cigarettes. JAMA Health Forum.

[6] Ratnapradipa K, Samson K, Dai HD (2023). Randomised experiment for the effect of 'tobacco-free nicotine' messaging on current e-cigarette users' perceptions, preferences and intentions. Tob Control.

[7] Camenga DR, Krishnan-Sarin S, Davis DR, Bold KW, Kong G, Morean ME (2022). Curiosity, use, and perceptions of "tobacco-free nicotine" e-cigarettes among U.S. young adults. Prev Med.

[8] Morean ME, Bold KW, Davis DR, Kong G, Krishnan-Sarin S, Camenga DR (2023). "Tobacco-free" nicotine pouches: risk perceptions, awareness, susceptibility, and use among young adults in the United States. Nicotine Tob Res.

[9] Center for Tobacco Products (2020). Enforcement priorities for electronic nicotine delivery system (ENDS) and other deemed products on the market without premarket authorization. U.S. Food and Drug Administration.

[10] Rostron BL, Cheng YC, Gardner LD, Ambrose BK (2020). Prevalence and reasons for use of flavored cigars and ENDS among US youth and adults: estimates from Wave 4 of the PATH study, 2016-2017. Am J Health Behav.

[11] Leventhal AM, Miech R, Barrington-Trimis J, Johnston LD, O'Malley PM, Patrick ME (2019). Flavors of e-cigarettes used by youths in the United States. JAMA.

[12] Schneller LM, Bansal-Travers M, Goniewicz ML, McIntosh S, Ossip D, O'Connor RJ (2019). Use of flavored e-cigarettes and the type of e-cigarette devices used among adults and youth in the US-results from Wave 3 of the Population Assessment of Tobacco and Health Study (2015-2016). Int J Environ Res Public Health.

[13] Lamb T, Muthumalage T, Meehan-Atrash J, Rahman I (2022). Nose-only exposure to cherry- and tobacco-flavored e-cigarettes induced lung inflammation in mice in a sex-dependent manner. Toxics.

[14] Omaiye EE, Luo W, McWhirter KJ, Pankow JF, Talbot P (2022). Ethyl maltol, vanillin, corylone and other conventional confectionery-related flavour chemicals dominate in some e-cigarette liquids labelled 'tobacco' flavoured. Tob Control.

[15] Pittet AO, Rittersbacher P, Muralidhara R (2002). Flavor properties of compounds related to maltol and isomaltol. J Agric Food Chem.

[16] Muthumalage T, Rahman I (2023). Pulmonary immune response regulation, genotoxicity, and metabolic reprogramming by menthol- and tobacco-flavored e-cigarette exposures in mice. Toxicol Sci.

[17] Eaton DL, Kwan LY, Stratton K, Stratton K, Kwan LY, Eaton Dl (2018). Chapter 5: toxicology of e-cigarette constituents. Public Health Consequences of E-Cigarettes.

[18] Tehrani MW, Newmeyer MN, Rule AM, Prasse C (2021). Characterizing the chemical landscape in commercial e-cigarette liquids and aerosols by liquid chromatography-high-resolution mass spectrometry. Chem Res Toxicol.

[19] Kaur G, Muthumalage T, Rahman I (2018). Mechanisms of toxicity and biomarkers of flavoring and flavor enhancing chemicals in emerging tobacco and non-tobacco products. Toxicol Lett.

[20] Yu V, Rahimy M, Korrapati A, Xuan Y, Zou AE, Krishnan AR, Tsui T, Aguilera JA, Advani S, Crotty Alexander LE, Brumund KT, Wang-Rodriguez J, Ongkeko WM (2016). Electronic cigarettes induce DNA strand breaks and cell death independently of nicotine in cell lines. Oral Oncol.

[21] Sundar IK, Javed F, Romanos GE, Rahman I (2016). e-Cigarettes and flavorings induce inflammatory and pro-senescence responses in oral epithelial cells and periodontal fibroblasts. Oncotarget.

[22] Kaur G, Gaurav A, Lamb T, Perkins M, Muthumalage T, Rahman I (2020). Current perspectives on characteristics, compositions, and toxicological effects of e-cigarettes containing tobacco and menthol/mint flavors. Front Physiol.

[23] Alshareef HZ, Omaye ST (2021). Toxicology of commonly found ingredients in e-cigarettes: a brief review. Health.

[24] Vaughan DE (2005). PAI-1 and atherothrombosis. J Thromb Haemost.

[25] Centers for Disease Control and Prevention (2019). e-Cigarette, or vaping, products visual dictionary. CDC Stacks.

[26] Williams M, Talbot P (2019). Design features in multiple generations of electronic cigarette atomizers. Int J Environ Res Public Health.

[27] Lu X, Chen L, Yuan J, Luo J, Luo J, Xie Z, Li D (2020). User perceptions of different electronic cigarette flavors on social media: observational study. J Med Internet Res.

[28] Gao Y, Xie Z, Li D (2022). Investigating the impact of the New York state flavor ban on e-cigarette-related discussions on Twitter: observational study. JMIR Public Health Surveill.

[29] Chen L, Lu X, Yuan J, Luo J, Luo J, Xie Z, Li D (2020). A social media study on the associations of flavored electronic cigarettes with health symptoms: observational study. J Med Internet Res.

[30] Luo J, Chen L, Lu X, Yuan J, Xie Z, Li D (2021). Analysis of potential associations of JUUL flavours with health symptoms based on user-generated data from Reddit. Tob Control.

[31] (2022). Monitoring U.S. e-cigarette sales: national trends. CDC Foundation.

[32] Shi H, Tavárez ZQ, Xie Z, Schneller L, Croft D, Goniewicz M, McIntosh S, O'Connor RJ, Ossip D, Rahman I, Li D (2020). Association of flavored electronic nicotine delivery system (ENDS) use with self-reported chronic obstructive pulmonary disease (COPD): results from the Population Assessment of Tobacco and Health (PATH) study, Wave 4. Tob Induc Dis.

[33] Department of Health and Human Services (2016). Deeming tobacco products to be subject to the Food, Drug, and Cosmetic Act, as amended by the Family Smoking Prevention and Tobacco Control Act; regulations restricting the sale and distribution of tobacco products and required warning statements for tobacco product packages and advertisements. Food and Drug Administration.

[34] (2019). Statement from FDA Commissioner Scott Gottlieb, M.D., on advancing new policies aimed at preventing youth access to, and appeal of, flavored tobacco products, including e-cigarettes and cigars. U.S. Food and Drug Administration.

[35] Xie Z, Ruan J, Jiang Y, Zhang B, Chen T, Luo J, Li D (2022). Potential impact of FDA flavor enforcement policy on vaping behavior on Twitter. Int J Environ Res Public Health.

[36] (2020). FDA finalizes enforcement policy on unauthorized flavored cartridge-based e-cigarettes that appeal to children, including fruit and mint. U.S. Food and Drug Administration.

[37] Sanchez S, Kundu A, Limanto E, Selby P, Baskerville NB, Chaiton M (2022). Smartphone apps for vaping cessation: quality assessment and content analysis. JMIR Mhealth Uhealth.

[38] Sassano MF, Davis ES, Keating JE, Zorn BT, Kochar TK, Wolfgang MC, Glish GL, Tarran R (2018). Evaluation of e-liquid toxicity using an open-source high-throughput screening assay. PLoS Biol.

[39] Krüsemann EJZ, Havermans A, Pennings JLA, de Graaf K, Boesveldt S, Talhout R (2021). Comprehensive overview of common e-liquid ingredients and how they can be used to predict an e-liquid's flavour category. Tob Control.

[40] Gralla EJ, Stebbins RB, Coleman GL, Delahunt CS (1969). Toxicity studies with ethyl maltol. Toxicol Appl Pharmacol.

[41] Jiang N, Xu S, Li L, Cleland CM, Niaura RS (2023). Use of electronic nicotine delivery system (ENDS) devices among U.S. youth and adults: findings from the Population Assessment of Tobacco and Health Study Waves 1-5. Addict Behav.

[42] Lin C, Baiocchi M, Halpern-Felsher B (2020). Longitudinal trends in e-cigarette devices used by Californian youth, 2014-2018. Addict Behav.

[43] Li D, Ossip DJ, Bansal-Travers M, Xie Z (2022). Impact of the FDA flavour enforcement policy on flavoured electronic cigarette use behaviour changes. Tob Control.

[44] Lindpere V, Winickoff JP, Khan AS, Dong J, Michaud TL, Liu J, Dai HD (2023). Reasons for e-cigarette use, vaping patterns, and cessation behaviors among US adolescents. Nicotine Tob Res.

[45] Landry RL, Groom AL, Vu TT, Stokes AC, Berry KM, Kesh A, Hart JL, Walker KL, Giachello AL, Sears CG, McGlasson KL, Tompkins LK, Mattingly DT, Robertson RM, Payne TJ (2019). The role of flavors in vaping initiation and satisfaction among U.S. adults. Addict Behav.

[46] Lu S, Liu S, Huang P, Wang Z, Wang Y (2021). Study on the combined toxicities and quantitative characterization of toxicity sensitivities of three flavor chemicals and their mixtures to Caenorhabditis elegans. ACS Omega.

[47] Pokhrel P, Herzog TA, Muranaka N, Regmi S, Fagan P (2015). Contexts of cigarette and e-cigarette use among dual users: a qualitative study. BMC Public Health.

[48] Azagba S, Ebling T, Shan L (2023). Sexual minority youth e-cigarette use. Pediatrics.

[49] Romm KF, Cohn AM, Wang Y, Williams R, Berg CJ (2023). Disparities in trajectories of cigarette and e-cigarette use across sexual orientation groups of young adult men and women in the US. Addict Behav.

[50] Sun Y, Lamb T, Prabhu P, Li D, McIntosh S, Rahman I ENDS Tobacco Flavors, Public Health, and Toxicity. Preprints.

